# Nonlinear Multiscale Entropy and Recurrence Quantification Analysis of Foreign Exchange Markets Efficiency

**DOI:** 10.3390/e20010017

**Published:** 2017-12-31

**Authors:** Hongli Niu, Lin Zhang

**Affiliations:** Donlinks School of Economics and Management, University of Science and Technology Beijing, Beijing 100083, China

**Keywords:** nonlinear analysis, complexity, exchange rate, MWPE approach, recurrence behaviors

## Abstract

The regularity of price fluctuations in exchange rates plays a crucial role in foreign exchange (FX) market dynamics. In this paper, we quantify the multiply irregular fluctuation behaviors of exchange rates in the last 10 years (November 2006–November 2016) of eight world economies with two nonlinear approaches. One is a recently proposed multiscale weighted permutation entropy (MWPE) and another is the typical quantification recurrence analysis (RQA) technique. Furthermore, we utilize the RQA technique to study the different intrinsic mode functions (IMFs) that represents different frequencies and scales of the raw time series via the empirical mode decomposition algorithm. Complexity characteristics of abundance and distinction are obtained in the foreign exchange markets. The empirical results show that JPY/USD (followed by EUR/USD) implies a a higher complexity and indicates relatively higher efficiency of the Japanese FX market, while some economies like South Korea, Hong Kong and China show lower and weaker efficiency of their FX markets. Meanwhile, it is suggested that the financial crisis enhances the market efficiency in the FX markets.

## 1. Introduction

Financial markets are some of the most complex systems that have been existed in human society, which exhibit rich fluctuation behaviors of financial price variations such as the fat tails phenomenon, power law of logarithmic returns and volumes, volatility clustering, multifractality of volatility, etc. Various models, techniques, and theoretical methods have been proposed to investigate the financial dynamics [1,2,3,4,5,6,7,8,9,10,11]. Owing to the great number of interactions among different agents, an almost arbitrary level of complexity of market segments is arrived upon. Thus, it is extremely meaningful and also natural that many methods from complex-system-theory and statistical sciences are more and more applied to analyze and depict these phenomena that are related to this sort of market [12,13,14,15,16]. In the present paper, we perform the nonlinear complex characteristics study of a certain market share, that is, the foreign exchange market (FX), which is the world’s largest and most liquid financial market and has a strong impact on the world economy, not only influencing the individual fortunes of billions but also directly affecting all other financial markets, since it manages the currencies trading and any price can be expressed in terms of a currency. Due to the liberalization of global economy, the international trade and flows of international capital significantly increase. Consequently, the supply and demand in the FX market experience violent changes, its risk rapidly grows, and the microeconomic and macroeconomic subject behaviors are dramatically influenced. The considerable growth of the trades and scales occur in the FX market, making the FX investment rank the second investment field of most importance. As a result, the risk management of the currency market correspondingly rises. Volatility in the foreign exchange market is influenced by numerous factors, like the exchange rate regimes followed by governments across the world and the transactions costs, which increasingly makes it of a a dynamic, complex, and unpredictable nature. Moreover, the complexity nature of FX market dynamic systems also comes from the existence of an independent frame of reference for currency pricing, i.e., any currency must be expressed in terms of a base currency, and from its sensitivity to interactions with all other financial markets. The occurrence, formation, and evolution of exchange rate fluctuations involve complex characteristics and exhibit nonlinear interactions, making it uniquely challenging in extracting any valuable information, forecasting and modeling.

Entropy, a concept from statistical physics, has become a powerful tool due to its capacity of capturing the time series’ uncertainty and disorder without exerting any restraints on the theoretical probability distribution [17]. A family of entropy parameters, such as Shannon entropy [18,19], Kolmogorov entropy [20], approximate entropy (ApEn) [21], sample entropy (SampEn) [22,23], etc. have witnessed extensive applications in various fields. Amongst them, permutation entropy was proposed by Bandt and Prompe [24], which is based on comparison of neighboring values of each point and mapping them to ordinal patterns. It shows advantages of simplicity, fast calculation, robustness for analyzing different kinds of time series, including random, chaotic and real-world time series, which has been employed in the context of neural [25], physiological signals [26,27,28], climate system [29] and financial market time series [30]. Despite its powerful ability in distinguishing dynamical behaviors of nonlinear time series, permutation entropy ignores the amplitude information. Thus, Fadlallah et al. introduced the weighted-permutation entropy (WPE) retaining the amplitude information of time series [31]. WPE permutes a vector in a time phase space and calculates the variance of the vector as a weight to compute the Shannon entropy, which can significantly enhance the robustness and stability of WPE, especially for the time series that contains substantial amplitude information because of its immunity to degradation by noise and (linear) distortion. Then, the multiscale weighted permutation entropy [32,33] was introduced as a combination of multiscale method and WPE, and was applied to the analysis of the actual signal series from vertical upward oil-in-water two-phase flow experiments. In this paper, we adopt the multiscale weighted permutation entropy (MWPE) approach to investigate complexity properties of exchange rates. Another approach we intend to use is recurrence plot (RP) and recurrence quantification analysis (RQA) [34,35,36,37]. RP provides visual insight into the complex nonlinear deterministic patterns hidden in a time series and shows a graphical description of recurrences that represent the similar system states obtained at different times. It portrays the distinct occasions when dynamical systems appear in the same region of phase space. RQA defines a group of recurrence measures that can be used to quantify important structures that the plot reveals. In particular, the RQA technique is further utilized in analysis of the IMF (intrinsic mode functions) series that represents various frequency scales of the original series after performing the empirical mode composition (EMD) [38] of price variations.

The rest of this paper is organized as follows. Section 2 describes the adopted data sets from the FX market. Section 3 introduces the multiscale weighted permutation entropy and the quantification recurrence analysis methods. In Section 4, the empirical complexity analysis results of price returns are presented, while Section 5 discusses the nonlinear recurrence performances of IMF time series. Finally, conclusions are drawn in Section 6.

## 2. Data Description and Processing

**Figure 1:**
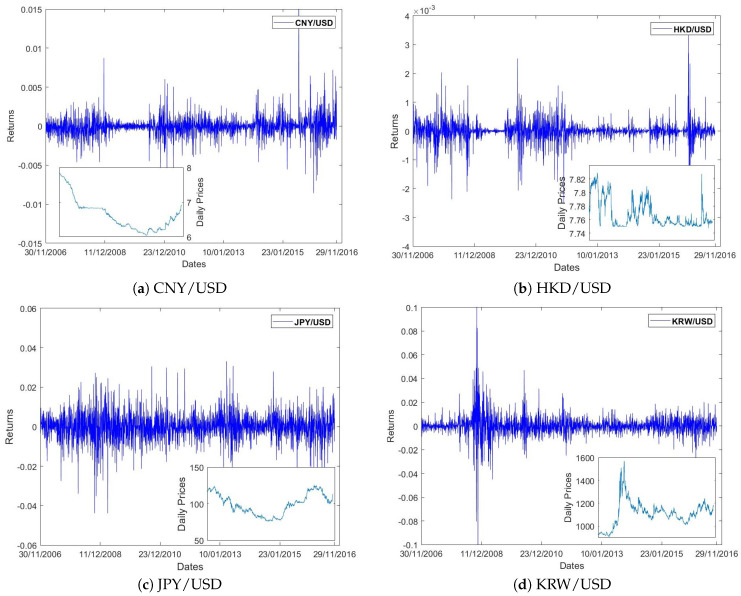

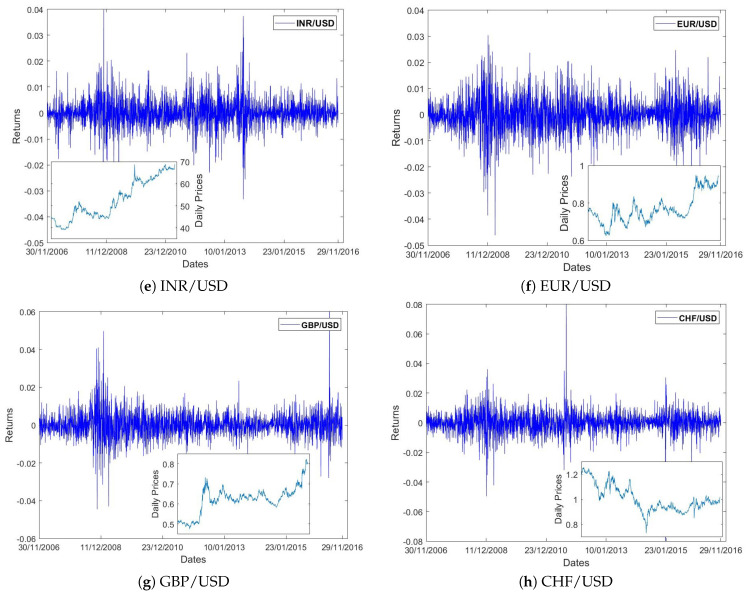
Daily logarithmic return time series of the exchange rates for eight world economies from 30 November 2006 to 29 November 2016. The inset plots show the daily price time series with the same time period.

The analyzed dataset consists of the exchange rates from eight different world economies, which are China renminbi yuan (CNY/USD), Hong Kong dollar (HKD/USD), Japanese yen (JPY/USD), South Korea won (KRW/USD), Indian Rupee (INR/USD), Euro (EUR/USD), U.K. pound sterling (GBP/USD) and Swiss franc (CHF/USD), respectively. The exchange rate is defined as local currency per US dollar, and covers the last 10-year time period from 30 November 2006 (just before the beginning of the financial crisis) to 29 November 2016, with 2457 data points. The nominal quotations are selected that are intended for statistical or analytical purposes Let $x_{t}$ denote the daily price of the exchange rate at day *t*. In this paper, we investigate the daily price changes (called logarithmic returns) of the exchange rate, which is calculated as its logarithmic difference, $r_{t}=\log x_{t}-\log x_{t-1}$. Figure 1 displays the time series graphs of daily prices and price returns of the exchange rates for eight world important economies, while Table 1 exhibits the descriptive statistics of the prices. The values of kurtosis for CNY/USD, HKD/USD, KRW/USD and GBP/USD are more than three (kurtosis >3= leptokurtic distribution) while the values for others are less than three (kurtosis <3= platykurtic distribution). These values clearly indicate the exchange rate data for the sample period is not normally distributed.

## 3. Methodologies

### 3.1. The MWPE Method

Permutation entropy (PE) [24] has been recently suggested as a complexity measure of nonlinear systems. It is based on the order relations among values of a time series, the permutation patterns. Though PE shows a number of advantages in distinguishing complex and dynamic properties of nonlinear time series, its ignorance of the amplitude information was pointed out [31], and correspondingly the weighted permutation entropy was developed by incorporating amplitude information. Later, the multiscale weighted permutation entropy (MWPE) was proposed by taking the multiple scales into consideration [32,33], which can be described as follows [33]:

(i) For a time series x(t)={x(1),x(t),⋯,x(N)}, its consecutive coarse-grained series, determined by the scale factor *s*, is constructed
  (1) $$y^{s}\left(t\right)=\frac{1}{s}\sum_{i=(j-1)s+1}^{js}x\left(t\right),\;1\leq t\leq N/s.$$
(ii) For $y^{s}$, given an embedding dimension *m* and a time delay η, an *m*-dimensional space is transformed from $y^{s}\left(t\right)$,
  (2) $$Y^{s}\left(t\right)=\left[y_{s}\left(t\right),y_{s}\left(t+\eta \right),\cdots ,y_{s}\left(t+\left(m-1\right)\eta \right)\right],\;1\leq t\leq N/s-m+1.$$
(iii) The components of $Y^{s}\left(t\right)$ are placed in an ascending order
  $$y^{s}\left(t+\left(k_{1}-1\right)\eta \right)\leq y^{s}\left(t+\left(k_{2}-1\right)\eta \right)\leq \cdots \leq y^{s}\left(t+\left(k_{m}-1\right)\eta \right).$$
  When confronting an equality, e.g.,
  $$y^{s}\left(t+\left(k_{i}-1\right)\eta \right)=y^{s}\left(t+\left(k_{j}-1\right)\eta \right)\left(i,j\in \left\{1,2,\cdots ,m\right\}\right),$$
  we consider the quantities *y* by the *k* values, namely if $k_{i}\leq k_{j}$, we set $y^{s}\left(t+(k_{i}-1\right)\eta )\leq y^{s}\left(t+(k_{j}-1\right)\eta )$. Thus, any vector $Y^{s}\left(t\right)$ has a permutation $\pi_{t}=\left[k_{1},k_{2},\cdots ,k_{m}\right]$, which is one of the permutations of *m* distinct symbol set {1,2,⋯,m}.
(iv) Of every permutation $\pi_{l}\left(l\in \left\{1,2,\cdots ,m!\right\}\right)$, the relative frequency with weight for $\pi_{l}$ is given as
  (3) $$p_{w}^{s}\left(l\right)=\frac{\sum_{t=1}^{N/s-(m-1)\eta }w\left(t\right)I_{l}\left(Y^{s}\left(t\right)\right)}{\sum_{t=1}^{N/s-(m-1)\eta }w\left(t\right)},$$
  where w(t) is the weighted value of $Y^{s}\left(t\right)$ and can be computed
  (4) $$w\left(t\right)=\frac{1}{m}\sum_{j=1}^{m}\left[y^{s}\left(t+\left(j-1\right)\eta \right)-{\bar{Y}}^{s}\left(t\right)\right],$$
  where ${\bar{Y}}^{s}\left(t\right)=\frac{1}{m}\sum_{j=1}^{m}y^{s}\left(t+\left(j-1\right)\eta \right)$ is the arithmetic mean of $Y^{s}\left(t\right)$. $I_{l}\left(Y^{s}\left(t\right)\right)$ is the indicator function of $Y^{s}\left(t\right)$ for permutation $\pi_{l}$, defined as $I_{l}\left(Y^{s}\left(t\right)\right)=1$ if $\pi_{t}=\pi_{l}$ and $I_{l}\left(Y^{s}\left(t\right)\right)=0$ if $\pi_{t}\neq \pi_{l}$.
(v) The MWPE $h_{w}^{s}$ is defined as the Shannon entropy
  (5) $$h_{w}^{s}=-\sum_{l=1}^{m!}p_{w}^{s}\left(l\right)\ln p_{w}^{s}\left(l\right).$$
  When $p_{w}^{s}\left(l\right)=1/m!$, then $p_{w}^{s}$ gets the maximum value ln(m!), thus $p_{w}^{s}$ can be normalized through ln(m!). The normalized MWPE $H_{w}^{s}$ is defined as $H_{w}^{s}=h_{w}^{s}/\ln\left(m!\right)$.

### 3.2. The RQA Approach

Recurrence quantification analysis (RQA) is a well-known nonlinear approach that is capable of investigating the complex deterministic properties of the dynamical systems. It offers numerical measures allowing for quantifying the structures and complexities that are embodied in the recurrence plot (RP) [34,35,36,37]. Considering a time series X(t), its phase space is constructed into the discrete time delay vector X(t)={X(t),X(t−η),X(t−2η),⋯,X(t−(m−1)η)}, where η denotes the time delay, and *m* is embedding dimension. RP emerges as a point in the phase space is approaching another point (at a distance lower than a certain threshold). Afterwards, the recurrence matrix R is given by combination of the Heaviside step function Θ(·) and the norm ||·||

(6) $$R_{ij}=\Theta \left(\varepsilon -||X\left(i\right)-X\left(j\right)||\right),\;i,j=1,2,\cdots ,N_{R},$$

where $N_{R}=N-\left(m-1\right)\eta$ is the number of considered states, and ε reflects the recurrence tolerance, called threshold value. R is comprised of zeros and ones corresponding to the state of the system (1—recurrence and 0—no recurrence).

Then, a number of complexity measures that are based on recurrence points’ densities, diagonal and vertical line structure [34,37] are provided in the RQA technique. The recurrence rate (RR)

(7) $$\mathrm{RR}=\frac{1}{N_{R}^{2}}\sum_{i,j=1}^{N_{R}}R_{i,j}$$

shows the density of recurrence points in an RP, and can be considered as the recurring probability of any state. The measure determinism (DET) comes from the line parallel to the main diagonal, defined by

(8) $$\mathrm{DET}=\frac{\sum_{l=l_{\min}}^{N_{R}}lP(l)}{\sum_{l=1}^{N_{R}}lP(l)},$$

where P(l) is a histogram of diagonal lines of the length *l*, and $l_{\min}$ is the minimal length of a diagonal line, which is set $l_{\min}=2$. DET offers an expression of determinism and predictability in the system, so the higher DET value is, the more predictable of the system with diagonal lines. Another measure defined for diagonal line collections is *Shannon entropy* $L_{\mathrm{ENT}}$

(9) $$L_{\mathrm{ENT}}=-\sum_{l=l_{\min}}^{N_{R}}p\left(l\right)\ln p\left(l\right),$$

where the probability of line distribution is $p\left(l\right)=P\left(l\right)/\sum_{l\geq l_{\min}}P\left(l\right)$. The increase of $L_{\mathrm{ENT}}$ suggests the rise complexity of the time series. Moreover, the mean length of the diagonal lines $L_{\mathrm{Mean}}=\sum_{l=l_{\min}}^{N_{R}}lp\left(l\right)$ is a measure that indicates the stability of the system. In the place of diagonal lines, the vertical recurrence lines are considered. Analogous to the determinism, the laminarity (LAM) is defined for vertical line patterns

(10) $$\mathrm{LAM}=\frac{\sum_{v=v_{\min}}^{N_{R}}vP(v)}{\sum_{v=1}^{N_{R}}vP(v)},$$

where P(v) denotes a histogram of vertical lines of the length *v* with the minimum line length $v_{\min}=2$. The larger value of the laminarity parameter reflects the the more stability of the system. Lastly, the average vertical line length, called “trapping time” $\mathrm{TT}=\sum_{v=v_{\min}}^{N_{R}}vp\left(v\right)$, measures the mean time that the system remains at a specific state.

## 4. Empirical Results of Price Returns

### 4.1. Complexity Analysis by the MWPE

**Figure 2:**
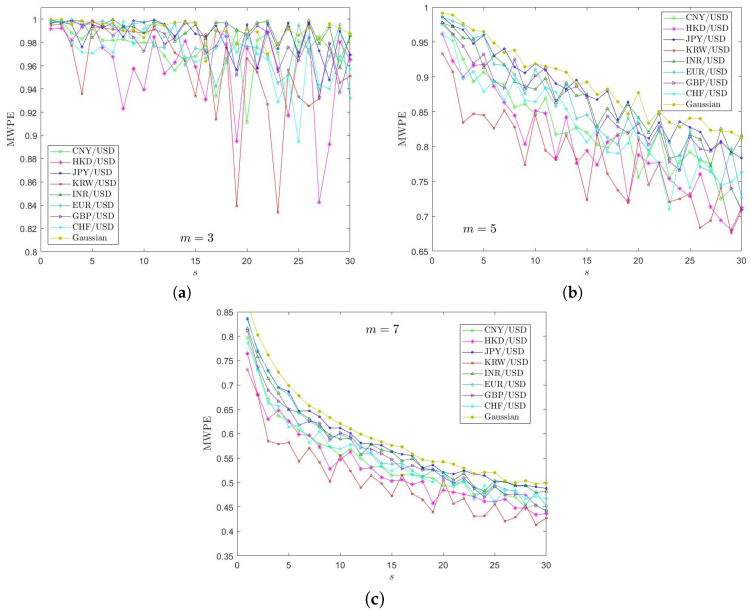
The multiscale weighted permutation entropy (MWPE) results for price returns of the exchange rates data when embedding dimension *m* is set 3 (**a**), 5 (**b**), 7 (**c**), respectively, and the time scale factor *s* varies from 1 to 30.

In this subsection, we apply the MWPE approach to investigate complexities of the price return series for the exchange rates introduced in Section 2. Figure 2 presents the empirical results of the MWPE analysis with time scale factor *s* from 1 to 30. In the MWPE algorithm, we choose the same time delay η=1 for all the analyzed time series. The embedding dimension *m* plays an important role in measurement of the permutation of probability distribution, since it determines the number of accessible states [39]. Bandt and Prompe [24] recommended m=3,4,⋯,7, and we compare the results for m=3,5,7, respectively. The influence of embedding dimension *m* on the estimation of MWPE values is very obviously observed in Figure 2. On one hand, when m=3, the MWPE values experience very significant oscillations for different scale factors, but with *m* increasing, the oscillations become less and less. In other words, the increase of *m* reduces the estimation errors of permutation entropies. On the other hand, the MWPE values become smaller with the *m* increasing but more stable. In practice, our applying of the MWPE analysis shows that increasing embedding dimension *m* beyond 6 affects indistinctly the tendency of obtained entropies values but greatly enlarges the running time. This to some extent provides the idea for choosing the embedding dimension of a nonlinear system. Table 2 clearly illustrates the average running time of one exchange rate series corresponding to various embedding dimension *m* from 3 to 9 when *s* ranging from 1 to 40. It is seen that the increasing of *m* will hugely raise the running time of the entropy algorithm.

We take the MWPE results when m=7 for analysis in Figure 2c, it is evident to observe a decreasing trend of MWPE values with the rise of scale factor *s*, indicating that the increasing of *s* can lead to the reducing complexity of local order structure of the price returns of all the analyzed exchange rates. The weighted permutation entropy values for JPY/USD on the whole scales are larger than those of other exchange rates and its curve is close to that of Gaussian data, implying a higher complexity of local order structure and indicating that the Japanese FX market is high efficient. This may be explained by its more flexibility of exchange rate regime. The MWPE curves of KRW/USD, HKD/USD fluctuate notably and deviate from the curve of Gaussian series, showing lower complexity properties of local order structures, which suggests that their market efficiencies are lower than the ones of other FX markets. To mention that the lower complexity of HKD/USD is quite understandable, since the HKD/USD is not a freely floating exchange rate and is essentially fixed against the USD through a currency board. Then, the CNY/USD follows. It is specially noticed that the INR/USD shares not very small MWPE values and is close to entropy values of EUR/USD and GBP/USD, suggesting a relatively not low market efficiency. Table 3 shows that the MWPE values of the exchange rates at different multiple scales *s*, which further illustrates the above empirical results.

**Figure 3:**
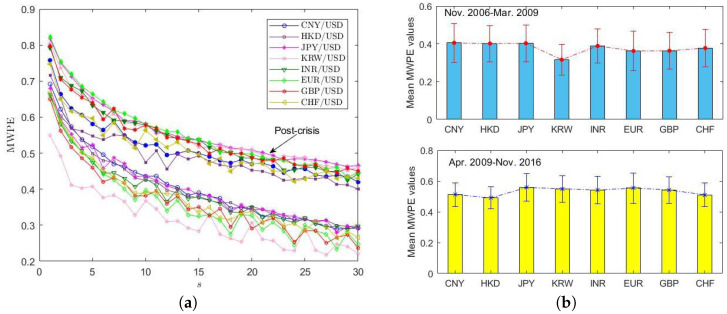
(**a**) Comparisons of the MWPE values obtained during the financial crisis and after the financial crisis for the exchange rates. (**b**) The mean MWPE values with error bars representing the standard deviation for 30 scale factors *s*.

In the following, we divide the data set into two periods: one is from November 2006 to March 2009 (during the financial crisis) and another is from April 2009 to November 2016 (post-crisis), and then perform the MWPE analysis on them with the parameters m=7 and η=1. Figure 3a shows the empirical results, from which the detailed changing and improvement of market efficiency can be observed. It is obvious that all the FX markets share larger MWPE values after financial crisis, displaying higher market efficiency. This illustrates that the financial crisis notably promotes the market efficiency in FX markets. Figure 3b manifests the mean values and error bars of MWPEs for the scale factors from 1 to 30 with regard to the two time periods. Once again, we see that the mean values of MWPE for each exchange rate data in post-crisis periods are larger than those in crisis periods. To have a clear view of the variation degrees of MWPE values between these two time periods, Table 4 presents the differences of the mean values and the MWPE values at scale factor s=20 for the exchange rates. It is seen from the table that, after the financial crisis, the market efficiency of the JPY/USD, the EUR/USD, the GBP/USD and the KRW/USD has improved significantly, which may be explained by one possible reason of high liquidity or trading volumes in the markets, especially after more than seven years of development. The variation degree of the HKD/USD is the smallest, then the CNY/USD FX market, followed by the CHF/USD and the INR/USD.

### 4.2. Determinism Analysis by the RQA

**Figure 4:**
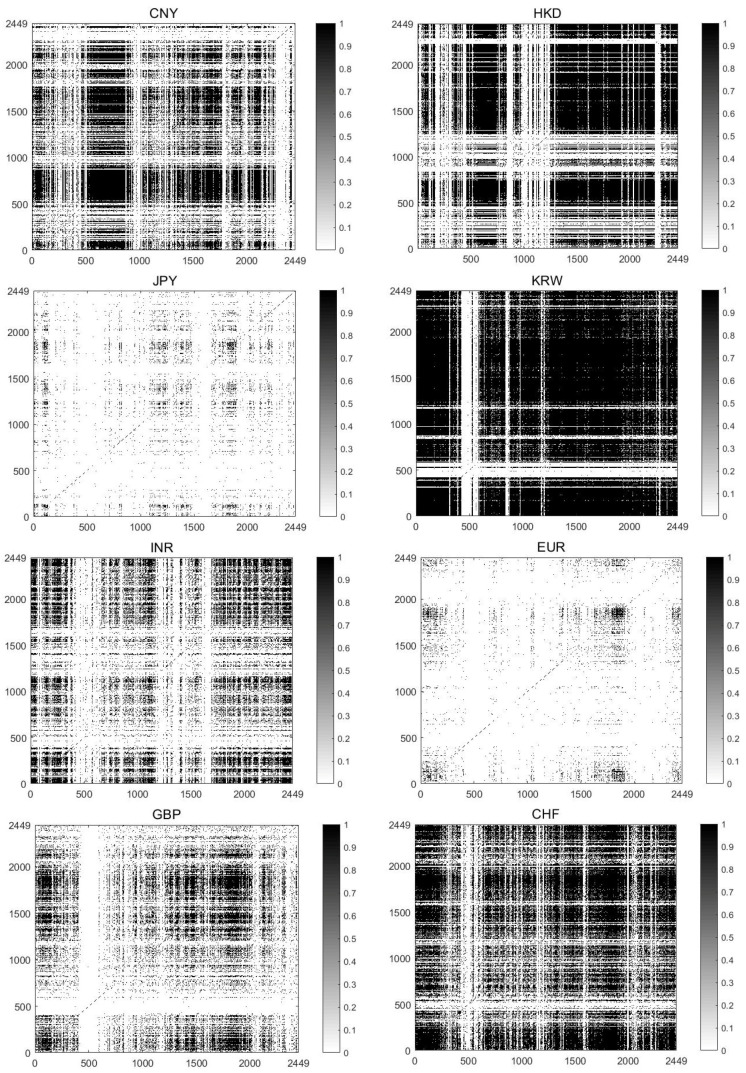
The recurrence matrix plots of the price returns for the eight analyzed exchange rates with m=8 and η=1, which are comprised of zeros and ones that correspond to the state of the system (1—recurrence and 0—no recurrence).

In the recurrence plot, it is significant to determine the time delay, embedding dimension and recurrence threshold. In the present paper, we apply the false nearest neighbors (FNNs) method [40] to calculate the embedding dimension (The optimal embedding is chosen as the one where the amount of the FNNs almost vanishes.), and m=8 seems to be suitable for all of the exchange rates. The time delay is fixed to η=1 by the average mutual information approach [41]. With regard to the recurrence tolerance ε, it is taken as approximately 10% of the maximal phase space diameter of price returns for each exchange rate data. Table 5 gives their ε values in performance of the recurrence analysis. It is observed that that the maximal phase space diameter of the HKD/USD (≅ 0.000852) is much smaller than others. The KRW/USD shares the largest maximal phase space diameter, followed by the CHF/USD. ε values of the JPY/USD, the INR/USD and the GBP/USD are around 0.01, while the values of the CNY/USD and the EUR/USD are smaller than 0.1. Figure 4 displays the recurrence plots of the exchange rate returns, which clearly visualize the recurrence properties of the dynamical system. In a recurrence plot, the dot at coordinate (i,j) is darkened if the distance ||X(i)−X(j)|| is smaller than a specified threshold. The recurrence structures roughly comprised of vertical and horizontal patterns and different distribution densities of recurrence points can be obviously observed. For example, the JPY/USD and the EUR/USD evidently have smaller recurrence density than others.

Table 5 shows the numerical calculations of the RQA measures for the exchange rates, providing a better quantification and understanding of the recurrences that are revealed in RP. It is noticeable that the RR value of the KRW/USD is much greater than other data, indicating the higher density of its recurrence points, which is also clearly revealed in Figure 4. The larger values of DET and LAM refer to the fraction of recurrence points forming diagonal lines and vertical lines for the KRW/USD being larger than those for the others, verifying that the KRW/USD is more deterministic and stable. $L_{\mathrm{ENT}}$ provides information as to the diversity of diagonal lines. The increasing $L_{\mathrm{ENT}}$ of the KRW/USD means a rise of the system’s complexity property. The larger TT measure suggests its longer mean time remaining at the specific state of the system. On the whole, the KRW/USD holds the highest values of RQA measure among these exchange rates, exhibiting a relatively stronger deterministic characteristic of the dynamic system of the returns. Following the KRW/USD are the HKD/USD, the CHF/USD (except RR value of the CHF/USD is smaller than the HKD/USD), the CNY/USD (except its TT values is larger than the CHF/USD), the INR/USD and the GBP/USD according to the values of RQA measures, and the JPY/USD and EUR/USD have the close but relatively smaller of the RQA measures, implying that their determinism properties are similar but relatively weaker among the analyzed data sets. This can illustrate relatively more efficiency of the EUR/USD and JPY/USD markets as one aspect, which may be explained by their market conditions, like high liquidity (A liquid market enhances competition among informed traders, and the speed of price discovery is much faster than in an illiquid market.) and information asymmetry (an excessive response by uninformed market makers to incoming order flows leads to weak predictive power of order imbalances in a high information asymmetry situation) [42].

## 5. Complexity Study of EMD-Based IMF Series

**Figure 5:**
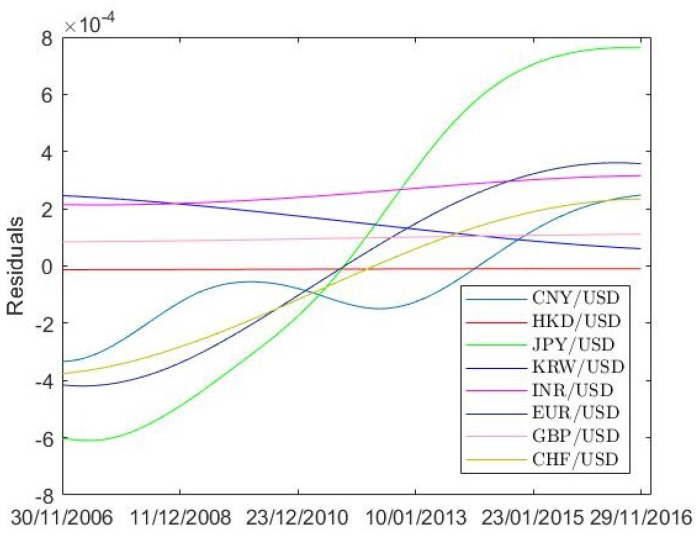
The trend components (the residual series through the empirical mode decomposition (EMD) decomposition) of the returns for the exchange rates during November 2006 and November 2016.

In this section, the MWPE approach is applied to investigate the determinism characteristics and recurrence behaviors of the exchange rates after performing the empirical mode decomposition (EMD) [38]. The essence of the EMD is to distinguish the intrinsic oscillatory modes, to empirically identify the local temporal and structural characteristic time scales in the data and then to decompose the data into the sum of a finite number of intrinsic mode functions (IMFs) and a final residual that expresses the trend fluctuations of the time series. With no pre-defined basis functions, IMFs are the time functions and make ideal for analyzing the non-stationary and nonlinear data. For more details about the EMD algorithm, see [38]. There are 10 IMF modes with one residual for the exchange rates. Figure 5 shows the residual from the EMD algorithm, which has been recognized as the trend of the given data. The first six IMFs are discussed in the present paper. Table 6 shows the embedding dimension *m* and time delay η in the phase space reconstruction. To save space, only the results for the EUR/USD are displayed, but IMFs of other exchange rates share the same embedding dimension and time delay with the EUR/USD. *m* value of the IMF1 is equal to that of the original ones (m=8 and m=9, respectively) while *m* of other IMFs are smaller (but *m* for IMF3 and IMF4 are the same). Every IMF holds evidently larger η value compared to that of the returns (η=1), especially for IMF5 and IMF6. About 10% of the maximal diameter of phase space is considered as the recurrence threshold ε. The calculation results of the EUR/USD are listed as a representative in Table 6. It is seen that ε values become gradually smaller from IMF1 to IMF6.

**Figure 6:**
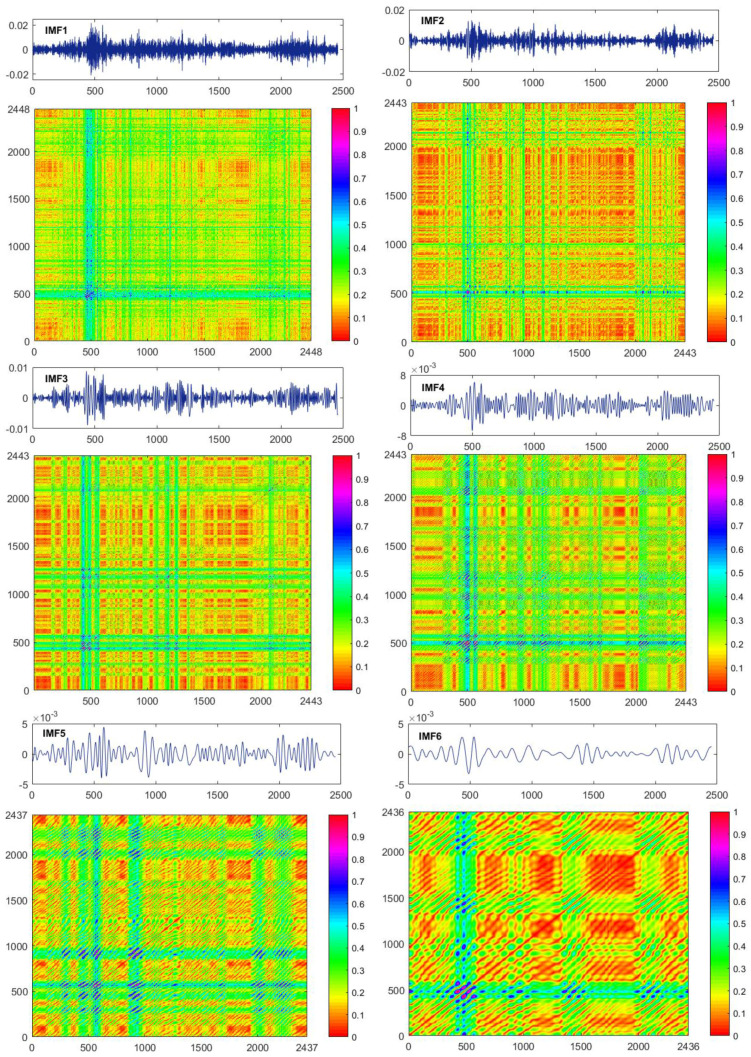
**Top**: the plots of the intrinsic mode function (IMF) series obtained from EMD decomposition of the exchange rate EUR/USD. **Bottom**: comparisons of recurrence distances of IMF1 to IMF6 for the EUR/USD returns.

Figure 6 describes the graphs of the first six IMF time series and their distance plots for the EUR/USD. RP depicts all the times at which a phase space trajectory visits roughly the same area in the phase space for a given moment in time. Its visual appearance brings insights into the dynamics of the system. Diverse and distinctive behaviors embodied in IMF systems are observable in the figure. IMF1 seems to share similarity of the recurrence pattern to that of the return series (see Figure 4), which may be explained by the fact that IMF1 holds the most information and properties of the original returns. Grid textures of recurrences consisting of vertical and horizonal lines for IMF1 to IMF5 can be observed. More and more visible recurrence points along the main diagonal lines are shown. Especially, IMF6 has quite a distinguishing recurrence pattern from the first five IMFs because of its sharing much more obvious recurrence points parallel to the main diagonal line. A great deal of information from the original returns are lost for the IMF4, IMF5 and IMF6, which is also revealed in their time series’ graphs.

Table 6 demonstrates the RQA results of the first six IMFs of the EUR/USD. IMF1 shows the lowest recurrence density (RR values), while IMF6 displays the highest one, which is also manifested in their RPs. In terms of the measures on diagonal recurrence lines, a significant increase of DET and $L_{\mathrm{Mean}}$ for IMF4, IMF5 and IMF6 is noticed in comparison with the first three IMFs, suggesting that the predictability time of the dynamical systems are longer. $L_{\mathrm{ENT}}$ shows information as to the diversity of diagonal lines and indicates the complex characteristics of the system. The larger $L_{\mathrm{ENT}}$ value of IMF6 suggests its higher complexity features. The IMF2 and IMF3 have approaching $L_{\mathrm{ENT}}$, implying close complexity. With regard to measures of of vertical recurrence lines, the very large LAM values from IMF3 to IMF6 illustrate their very high fractions of recurrence points in the vertical lines, representing that their systems are more stable in contrast with IMF1 and IMF2. Similar to $L_{\mathrm{Mean}}$, IMF5 and IMF 6 display a dramatic increase of the TT measures, which reflects the longer mean time for the system to remain at a specific state and further the stability of the system. Generally, IMF6 distinctly shares the highest values of overall RQA measures among these IMFs, which means that the determinism property of IMF6 is the strongest and most significant.

**Figure 7:**
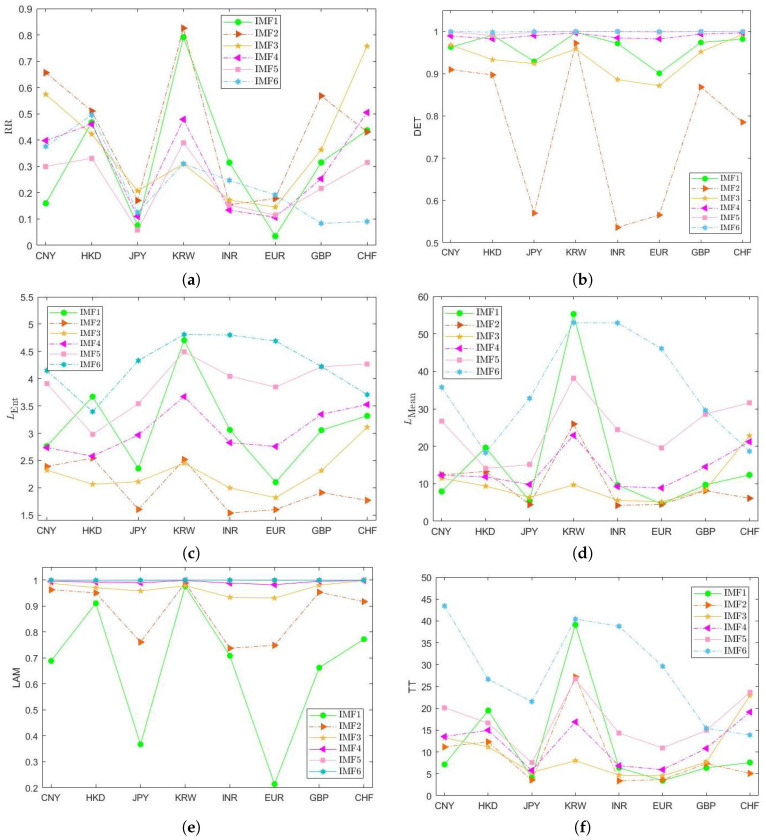
Comparisons of RQA measures ((**a**) recurrence rate (RR), (**b**) determinism (DET), (**c**) $L_{\mathrm{ENT}}$, (**d**) $L_{\mathrm{Mean}}$, (**e**) laminarity (LAM) and (**f**) trapping time (TT)) of IMF1 to IMF6 series obtained from the EMD decomposition of the exchange rate returns.

Figure 7 fully displays the variations of the RQA parameters, in which a comparison analysis of the fluctuation behaviors of overall time series dynamical systems is made. For instance, with regard to the $L_{\mathrm{Mean}}$ value of IMF1, the KRW/USD is markedly the largest (55.2825), followed by the HKD/USD (19.6320) and CHF/USD (12.3546). Then, the INR/USD and the GBP/USD are close, and are larger than CNY/USD. The EUR/USD is the lowest and very close to the JPY/USD, indicating less stability and determinism of their FX markets.

## 6. Conclusions

This paper intends to explore the nonlinear complexity properties of foreign exchange markets, in which eight exchange rates from eight important world economies are selected. Firstly, in the multiscale weighted permutation entropy analysis, it is observed that the MWPE values have very intensive oscillations when m=3 at different scale factors, but an increase of *m* can reduce the estimation errors of permutation entropies despite its greatly lengthening the running time. Among the exchange rates studied, the JPY/USD implies a higher complexity of local order structure and indicates higher efficiency of the Japanese FX market. The KRW/USD, HKD/USD show relatively lower complexity properties of local order structures, which suggests that their market efficiencies are lower than the ones of other FX markets, following the CNY/USD. Moreover, the comparisons study of MWPE values for the crisis and post-crisis periods shows that the market efficiency of FX markets is notably promoted after the financial crisis, especially for the JPY/USD, the EUR/USD, the GBP/USD and the KRW/USD. The HKD/USD displays the lowest degree of variation of the market efficiency. In Section 4, we utilize the RQA approach to discuss the complex determinism properties of the exchange rates. From the empirical results, the KRW/USD shows a relatively stronger determinism property of the dynamic system, followed by the HKD/USD, the CHF/USD, and the CNY/USD. The JPY/USD and EUR/USD imply similar and weaker determinism properties, which suggests that their FX markets are less predictable and more efficient. Through the EMD decomposition of the exchange rates in Section 5, diverse and distinctive nonlinear deterministic characteristics of IMFs that represent different scales and frequencies of returns being revealed. From IMF1 to IMF6, information held in the original series gradually lose and the system becomes more stable and predictable.

## Figures and Tables

**Table 1:** Descriptive statistics of daily prices of the exchange rate data.

Symbol	Mean	Std.	Min.	Median	Max.	Mode	Kur.	Skew.
CNY/USD	6.6241	0.4487	6.0409	7.8346	6.8276	6.8276	3.3777	1.0076
HKD/USD	7.7677	0.0202	7.7474	7.8288	7.7501	7.7501	3.2079	1.1966
JPY/USD	99.5427	14.3627	75.7186	100.0821	125.5933	76.6822	1.8443	0.0441
KRW/USD	1111.5366	108.0174	903.8059	1111.5366	1570.0365	923.9899	4.8571	0.6416
INR/USD	52.7574	8.9675	39.1129	50.2955	68.8737	39.1667	1.6737	0.2335
EUR/USD	0.7682	0.0753	0.6246	0.7521	0.9503	0.7552	2.7133	0.6841
GBP/USD	0.6206	0.0669	0.4738	0.6288	0.8228	0.6240	3.4634	−0.1852
CHF/USD	1.0042	0.1037	0.7299	0.9755	1.2535	0.9349	2.6815	0.6531

**Table 2:** Running time (seconds) of the multiscale weighted permutation entropy (MWPE) method performed on one piece of exchange rate data.

m=3	m=4	m=5	m=6	m=7	m=8	m=9
0.31337	0.70738	2.80653	16.02005	113.01158	928.67353	8421.36806

**Table 3:** MWPE values of price reruns of the exchange rates and Gaussian data at different scale factors *s* when m=7.

	s=1	s=3	s=5	s=7	s=9	s=11	s=13	s=15	s=17	s=20
CNY/USD	0.7967	0.6696	0.6272	0.5962	0.5731	0.5682	0.5318	0.5145	0.5164	0.4932
HKD/USD	0.7645	0.6302	0.6258	0.5966	0.5280	0.5627	0.5301	0.5033	0.4960	0.4844
JPY/USD	0.8363	0.7298	0.6865	0.6467	0.6118	0.6008	0.5791	0.5638	0.5550	0.5207
KRW/USD	0.7316	0.5850	0.5818	0.5705	0.5016	0.5233	0.5138	0.4725	0.4767	0.5048
INR/USD	0.8161	0.7132	0.6486	0.6304	0.5970	0.5904	0.5778	0.5627	0.5486	0.5201
EUR/USD	0.8342	0.7286	0.6808	0.6255	0.5916	0.5895	0.5610	0.5377	0.5239	0.5204
GBP/USD	0.8114	0.6895	0.6508	0.6255	0.5874	0.5940	0.5679	0.5471	0.5348	0.5103
CHF/USD	0.7863	0.6622	0.6136	0.5816	0.5732	0.5777	0.5581	0.5237	0.5151	0.5160
Gaussian	0.8678	0.7618	0.6987	0.6571	0.6329	0.6099	0.5909	0.5759	0.5582	0.5423

**Table 4:** Differences of MWPE values between post-crisis and crisis periods for the exchange rate data.

	CNY/USD	HKD/USD	JPY/USD	KRW/USD	INR/USD	EUR/USD	GBP/USD	CHF/USD
mean	0.1069	0.0884	0.1549	0.2335	0.1520	0.1924	0.1783	0.1327
s=20	0.1270	0.1003	0.1661	0.1995	0.1364	0.1709	0.1975	0.1316

**Table 5:** Recurrence quantification analysis (RQA) parameters of the price returns for the exchange rates. RR: recurrence rate; DET: determinism; LAM: laminarity; TT: trapping time.

Data			ε	RR	DET	$L_{\mathrm{ENT}}$	$L_{\mathrm{Mean}}$	LAM	TT
CNY/USD			0.0030	0.3439	0.9665	2.9517	9.4494	0.8653	8.4481
HKD/USD			0.000852	0.4713	0.9842	3.4302	15.9962	0.9457	16.5285
JPY/USD			0.0104	0.0346	0.8471	1.8694	3.9859	0.4149	3.0828
KRW/USD			0.0280	0.7529	0.9955	4.2750	35.8296	0.9784	25.2570
INR/USD			0.0125	0.2467	0.9499	2.6748	7.0894	0.7608	5.3144
EUR/USD			0.0099	0.0319	0.8329	1.8931	4.0782	0.4170	3.1038
GBP/USD			0.0147	0.2094	0.9336	2.5286	6.3789	0.6928	4.6615
CHF/USD			0.0229	0.5309	0.9746	3.2140	11.6567	0.8779	7.9887

**Table 6:** RQA parameters of the intrinsic mode functions (IMFs) of the price returns for the EUR/USD.

Data	*m*	η	ε	RR	DET	$L_{\mathrm{ENT}}$	$L_{\mathrm{Mean}}$	LAM	TT
IMF1	8	1	0.0079	0.0347	0.9008	2.1014	4.6083	0.2144	3.4507
IMF2	5	3	0.0047	0.1781	0.5657	1.5994	4.5146	0.7487	3.6681
IMF3	4	4	0.0025	0.1454	0.8715	1.8186	5.1570	0.9308	4.5780
IMF4	3	6	0.0016	0.1069	0.9823	2.7571	8.8940	0.9822	5.9583
IMF5	3	9	0.0011	0.1148	0.9987	3.8455	19.6199	0.9982	10.9299
IMF6	2	19	0.000708	0.1917	0.9999	4.6898	46.0817	0.9999	29.6150
